# Single‐Cell Sequencing Reveals Functional Alterations in Tuberculosis

**DOI:** 10.1002/advs.202305592

**Published:** 2024-01-08

**Authors:** Mengyuan Lyu, Gaolian Xu, Jian Zhou, Julien Reboud, Yili Wang, Hongli Lai, Yi Chen, Yanbing Zhou, Guiying Zhu, Jonathan M. Cooper, Binwu Ying

**Affiliations:** ^1^ Department of Laboratory Medicine West China Hospital Sichuan University Chengdu Sichuan 610041 P. R. China; ^2^ School of Biomedical Engineering/Med‐X Research Institute Shanghai Jiao Tong University Shanghai 200030 P. R. China; ^3^ Department of Thoracic Surgery West China Hospital Sichuan University Chengdu Sichuan 610041 P. R. China; ^4^ Division of Biomedical Engineering University of Glasgow Glasgow G12 8LT United Kingdom

**Keywords:** functional variation, heterogeneity, immunosuppression, single‐cell sequencing, tuberculosis

## Abstract

Despite its importance, the functional heterogeneity surrounding the dynamics of interactions between mycobacterium tuberculosis and human immune cells in determining host immune strength and tuberculosis (TB) outcomes, remains far from understood. This work now describes the development of a new technological platform to elucidate the immune function differences in individuals with TB, integrating single‐cell RNA sequencing and cell surface antibody sequencing to provide both genomic and phenotypic information from the same samples. Single‐cell analysis of 23 990 peripheral blood mononuclear cells from a new cohort of primary TB patients and healthy controls enables to not only show four distinct immune phenotypes (TB, myeloid, and natural killer (NK) cells), but also determine the dynamic changes in cell population abundance, gene expression, developmental trajectory, transcriptomic regulation, and cell–cell signaling. In doing so, TB‐related changes in immune cell functions demonstrate that the immune response is mediated through host T cells, myeloid cells, and NK cells, with TB patients showing decreased naive, cytotoxicity, and memory functions of T cells, rather than their immunoregulatory function. The platform also has the potential to identify new targets for immunotherapeutic treatment strategies to restore T cells from dysfunctional or exhausted states.

## Graphical abstract

Based on single‐cell RNA sequencing and cell surface antibody sequencing, the immune atlas of tuberculosis is described. The naive, cytotoxicity, and memory functions of blood T cells from tuberculosis patients are suppressed, while the immunoregulatory function is not. These findings highlight the potential of immunotherapy strategies for restoring T cell dysfunction or exhaustion in anti‐tuberculosis therapy.

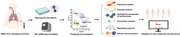

## 1 Introduction

Tuberculosis (TB) is a leading cause of death globally, highlighting the urgent need for a more complete understanding of the pathogenic mechanism of *Mycobacterium tuberculosis* (MTB) and the nature of its interactions with the human immune response. It is known that during infection, MTB exports its proteins, transporting them into the host cells,^[^ ^1^ ^]^ while at the same time, the host recognizes pathogen‐associated molecular patterns and initiates a cascade of interferon (IFN)‐γ production and phagolysosomal acidification (a response which also includes the production of reactive oxygen and nitrogen species).^[^ ^2^ ^]^ In turn, MTB interferes with the host's immune defense, for example by releasing lipoarabinomannan to inhibit the maturation and acidification of the host's phagolysosome, whilst also blocking the powerful oxidative action of the host's reactive oxygen and nitrogen species.^[^ ^3^ ^]^

The understanding of these “back‐and‐forth” interactions and the molecular pathways involved has led to insights into currently unresolved mechanisms by which MTB can survive.^[^ ^4^ ^]^ Despite this knowledge, the functional heterogeneity surrounding the dynamics of the interactions between MTB and human immune cells and its role in determining host immune strength and TB outcomes, remains less well understood, with infected patients developing different levels of immune responses, with divergent clinical symptoms or radiographic features and with different disease outcomes.^[^ ^5^ ^]^ Challenges in both understanding and interpreting the numerous cellular and molecular interactions underpinning this heterogeneity have led to investigations of the determinants of that diversity.^[^ ^6^ ^]^

One technique previously used to study the molecular mechanisms of heterogeneity, at the resolution of a single cell involves single‐cell RNA sequencing (scRNA‐seq).^[^ ^7^ ^]^ Analysis of transcription profiles in TB patients has provided insights into the abundance and state of specific cell sub‐populations at the onset of the disease.^[^ ^8^ ^]^ ScRNA‐seq has also revealed functional differences between tissues locally affected by MTB, which have been considered as a molecular switch that controls the course of the infection,^[^ ^9^ ^]^ and as a marker of disease progression.^[^ ^10^ ^]^

Given that communication with and between immune cells, including through cytokines and chemokines, carries critical information about TB, here, we integrated analysis of scRNA‐seq with cell surface antibody sequencing (AbSeq) to systematically decode the functional variation of peripheral blood mononuclear cells (PBMCs) from primary TB patients and healthy controls (HCs). Our results not only show that TB patients have T‐cell dysfunction that may be realized by altering gene expression profiling, cell abundance, and cellular communication. Our results also identify that the exhaustion of T cells may be a key outcome of TB, which could suggest new immunotherapeutic strategies for TB treatment.

## 2 Results

### 2.1 TB‐Related Cellular and Transcriptomic Landscape at the Single‐Cell Level

**Figure 1:**
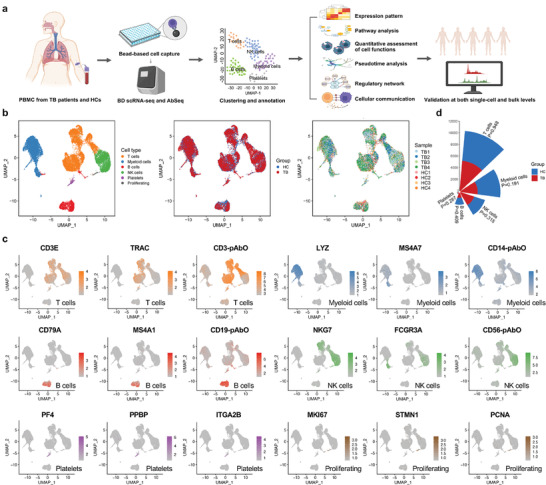
Single‐cell profiling of eight PBMC samples from four primary TB patients and four HCs based on scRNA‐seq and AbSeq. a) Experimental and analytic flow. b) UAMP plots of 23 990 individual cells from eight participants colored by cell type, group, and sample, respectively. c) Feature plots showing the expression level of marker genes for six PBMC subsets. The gray color indicated the genes were not expressed or were expressed at a relatively low level in the corresponding cells. Molecules with names ending with pAbO represent cell surface proteins. d) Nightingale rose plot showing the number of five PBMC subsets colored by the group. The height of each bar graph represented the mean number of each cell subtype across the four samples in the HC or TB group. *p* values are computed from a two‐sided Student's *t‐*test. Abbreviations: PBMC, peripheral blood mononuclear cell; TB, tuberculosis; HC, healthy control; scRNA‐seq, single‐cell RNA sequencing; AbSeq, antibody sequencing.

A total of eight participants, four primary TB patients, and four HCs, were enrolled in the first study cohort (**Figure** **1**a; Table S1, Supporting Information). ScRNA‐seq data from 11 611 cells of TB patients and 12 379 cells from HCs entered the downstream analysis. After PCA and UMAP analysis, clustering analysis based on a shared nearest neighbor (SNN) modularity optimization categorized these 23 990 cells into 17 distinct clusters. Using well‐labeled marker genes and the results of AbSeq, the 17 clusters were further grouped into T cells (*CD3*, *CD4*, *CD8* and *TRAC*), myeloid cells (*LYZ*, *S100A9*, *MS4A7* and *CD14*), B cells (*CD19* and *CD79A*), natural killer (NK) cells (*CD16* and *CD56*), platelets (*PPBP* and *MKI67*) and proliferating cells (*STMN1*) (Figure 1b,c; Table S2, Supporting Information). Among the above six types, T cells accounted for the largest proportion in both groups (42.63% in TB patients and 42.21% in HCs), followed by myeloid cells (25.84% and 31.58%, respectively). The proportions of these six types in these two groups were not statistically significant (Figure 1d). The cell composition of those individuals included is shown in Figure S1 (Supporting Information).

### 2.2 Deciphering the Heterogeneity of T Cells in TB

**Figure 2:**
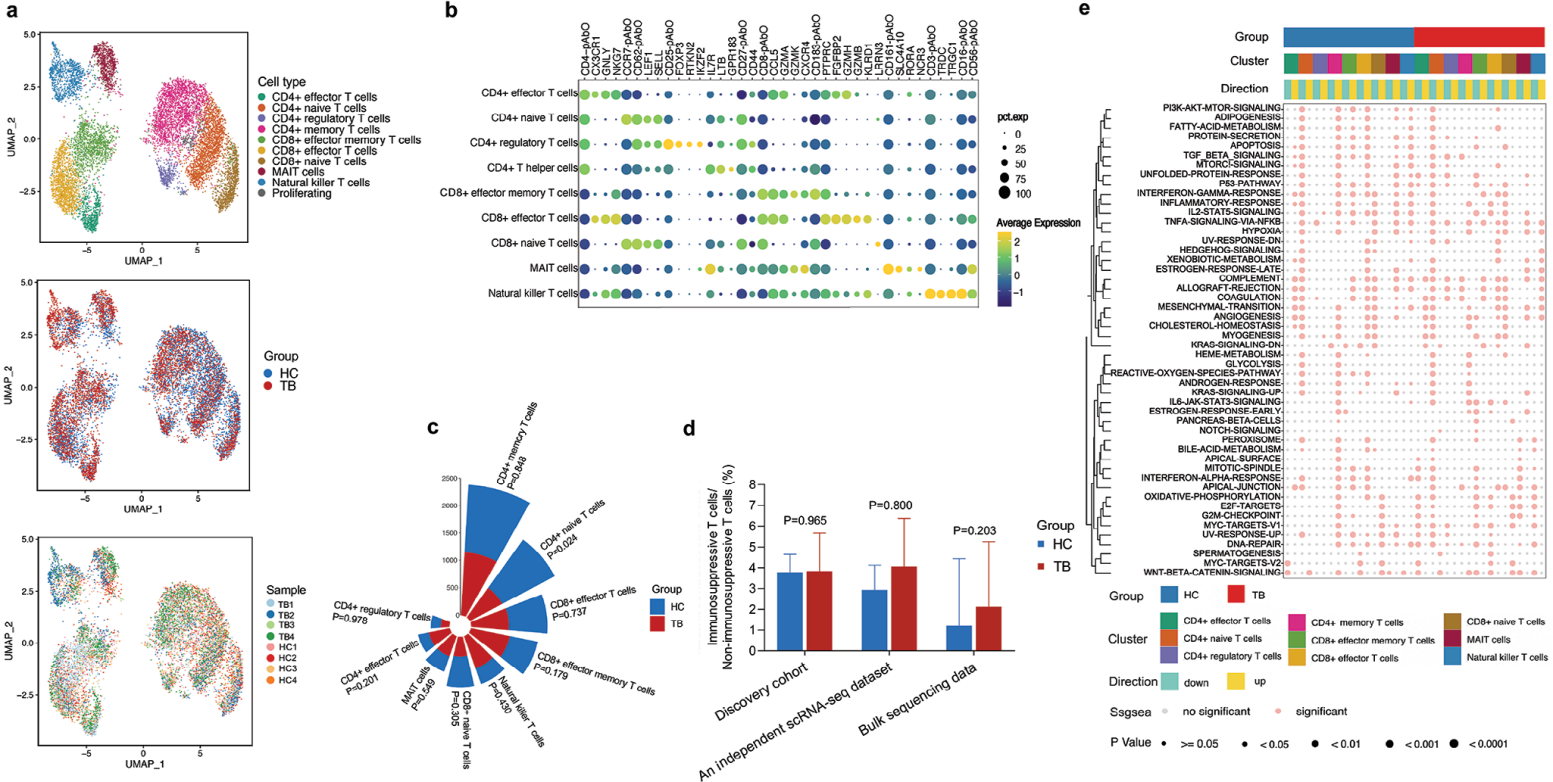
Cluster and cell subtype analysis of T cells. a) UMAP plots of 10 371 T cells colored by cell type, group, and sample (top to bottom). b) Dot plot for expression of marker genes for nine T cell subtypes. Molecules with names ending with pAbO represent cell surface proteins. Red boxes represented hallmark genes for each T cell subtype. c) Nightingale rose plot showing a number of nine T cell subtypes colored by the group. P values are computed from a two‐sided Student's *t*‐test. d) Histogram showing the ratio of immunosuppressive T cells (CD4+ regulatory T cells) and non‐immunosuppressive T cells (CD4+ naive T cells, CD8+ naive T cells, CD4+ memory T cells, CD4+ effector cells, CD8+ effector T cells, CD8+ effector memory T cells, MAIT cells, and NKT cells) in the TB and HC group. P values are computed from a two‐sided Student's *t*‐test. e) Differences in ssGSEA score of hallmark top 50 gene sets in T cell subtypes between TB and HC group. The color and size of the dots together indicate enrichment significance. *p* values are computed from the Wilcox test. Abbreviations: MAIT, mucosal‐associated invariant T; HC, healthy control; TB, tuberculosis; ssGSEA, single‐cell rank‐based gene set enrichment analysis.

A second cell clustering for T cells at a more granular level was used to dissect the behaviors of their subtypes in TB. Eleven clusters from 10 371 T cells were categorized into four CD4+ T cell subsets, three CD8+ T cell subsets, two atypical T cell subsets, and a subset of proliferating cells (**Figure** **2**a). CD4+ T cells were composed of CD4+ naive T cells (*CCR7* and *CD62L*), CD4+ memory T cells (*IL7R* and *LTB*), CD4+ regulatory T cells (*FOXP3* and *RTKN2*) and CD4+ effector T cells (*CX3CR1* and *GNLY*).^[^ ^11^ ^]^ CD8+ T cells included CD8+ naive T cells (*CCR7* and *LRRN3*), CD8+ effector memory T cells (*CCL5*, *GZMA*, and *GZMK*), and CD8+ effector T cells (*FGFBP2* and *GNLY*). Two atypical T cell subsets were mucosal‐associated invariant T (MAIT) cells (*CD161* and *SLC4A10*) and natural killer T (NKT) cells (*CD3*, *CD16*, *CD56*) (Figure 2b; Table S3, Supporting Information).

Naive T cells were the main T cell subtype whose abundance was reduced in TB patients, with the proportion of CD4+ naive T cells in the TB group being significantly lower than that in the HC group (*p* = 0.024) (Figure 2c). The abundances of some effector T cells (CD4+ effector T cells, CD8+ effector memory T cells, etc.) were also increased in TB patients, as was also reported previously.^[^ ^12^ ^]^ Most importantly, we found that the TB group had a higher ratio of immunosuppressive (CD4+ regulatory T cells) and non‐immunosuppressive T cells (other T cell subtypes) than the HC group (Figure 2d), although the difference did not reach significance. Results in an independent scRNA‐seq dataset and bulk sequencing data (Table S4 and Figure S2, Supporting Information) also support this finding.

Enrichment analysis further showed that inflammation‐related pathways were significantly suppressed in TB patients (Figure 2e), primarily confirming the potential for MTB to disrupt host immune balance and tilt it toward immunosuppression. For instance, IFN‐α response of CD4+ effector T cells, CD8+ effector memory T cells, CD8+ effector T cells, and MAIT cells were suppressed in TB patients. The IFN‐γ response ability of CD4+ naive T cells, CD4+ regulatory T cells, and CD4+ memory T cells in TB patients was also impaired. Crawford et al.^[^ ^13^ ^]^ found that the functional CD4+ T cell response was essential to avoid CD8+ T cell exhaustion during infections. We speculated that the CD8+ T cell dysfunction or exhaustion could be triggered by interfering with the ability of CD4+ T cells to respond to IFN‐γ. This triggering mechanism was also reflected by increased *LAG3* expression of CD8+ effector cells and CD8+ effector memory cells in the TB group (Table S5, Supporting Information). *LAG3* expression in MAIT cells and NKT cells was also higher in the TB group than in the HC group. It should be noted that we did not observe the significant co‐expression of *LAG3* and *PD‐1* that is often seen in other diseases (Table S5, Supporting Information).^[^ ^14^ ^]^

### 2.3 Quantitatively Evaluating TB‐Related Function Variations in T Cells

**Figure 3:**
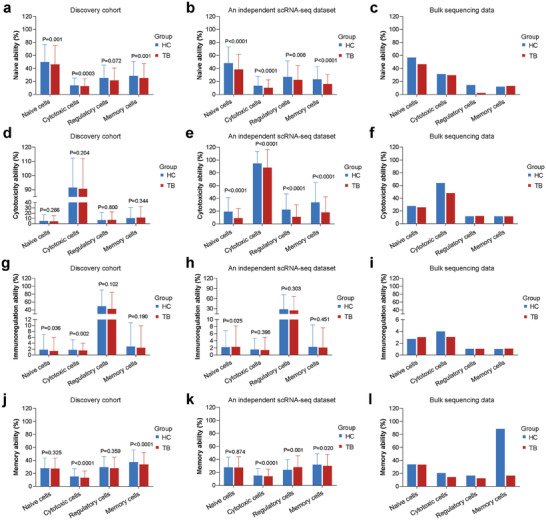
Differences in functional abilities of four T cell subsets between TB and HC groups based on the discovery cohort (a, d, g, j), an independent scRNA‐seq dataset (b, e, h, k) and bulk data (c, f, j, l), in columns. Nine T cell subtypes were clustered into four categories according to their function. Naive cells included CD4+ naive T cells and CD8+ naive T cells. Cytotoxic cells included CD8+ effector T cells, CD8+ effector memory T cells, CD4+ effector T cells, MAIT cells, and NKT cells. Regulatory cells are referred to as CD4+ regulatory T cells and memory cells are referred to as CD4+ memory T cells. (in rows) Differences in naive a–c), cytotoxicity d–f), immunoregulation g–i), and memory j–l) functional ability of four T cell subsets between TB and HC groups. *p* values are computed from a two‐sided Student's *t*‐test. It should be noted that the deconvolution results in the bulk data did not support the calculation of *p* values.

We further classified all T cells into naive, cytotoxic, regulation, and memory cells. The expression of the selected genes represented corresponding functions are shown in **Table** **1** and Figure S3 (Supporting Information). Naive cells in the TB group exhibited lower naive function than those in the HC group (*p* = 0.001) (**Figure** **3**a), which was again validated by the independent scRNA‐seq dataset (*p* < 0.0001) (Figure 3b) and bulk analysis (Figure 3c). Cytotoxic cells in the TB group harbored decreased cytotoxicity ability compared with those in the HC group in all datasets (Figure 3d–f). However, such difference did not reach significance in our discovery cohort (*p* = 0.204), but not in the scRNA‐seq dataset (*p* < 0.0001). There was no significant difference in the immunoregulatory ability of regulatory cells between TB and HC groups in any datasets (Figure 3g–i). The memory ability of memory cells in the TB group was diminished in all datasets (Figure 3j–l). And significant differences were observed in both our discovery cohort (*p* < 0.0001) and the scRNA‐seq dataset (*p* = 0.020). Taken together, these results indicate that TB patients had decreased immune functions that contributed positively to the host immune strength (naive, cytotoxicity, and memory function), but not the function that negatively affected the host immune strength (immunoregulation function).

**Table 1:** Representative cell subtypes and the gene set of each T cell function.

Function	Representative T cell subtype	Representative gene	Functional gene set
Naive	CD4+ naive CD8+ naive	*CCR7*	*CCR7*, *RPS14*, *EEF1A1*, *RPS28*, *HLA‐E*, *RPL29*, *RPL13*, *RPS18*, *RPS19*, *RPS7*
Cytotoxicity	MAIT^a)^ Natural killer CD4+ effector CD8+ effector CD8+ effector memory	*GZMA*	*GZMA*, *ACTB*, *B2M*, *TMSB4X*, *PFN1*, *HLA‐C*, *CFL1*, *HLA‐A*, *NKG7*, *HLA‐B*
Immunoregulation	CD4+ regulatory	*FOXP3*	*FOXP3*, *TRAC*, *SMCHD1*, *CTSS*, *ELK3*, *IL32*, *TMEM248*, *TAF1*, *CD4*, *CD68*
Memory	CD4+ memory	*LTB*	*LTB*, *HLA‐B*, *RPS28*, *RPL23*, *RPS19*, *RPL7*, *HLA‐A*, *RPS29*, *MT‐ATP6*, *MT‐ND5*

^a)^ mucosal‐associated invariant T

### 2.4 Tracing the Developmental Trajectories of Cells and the Expression Patterns of Representative Genes in T Cells

**Figure 4:**
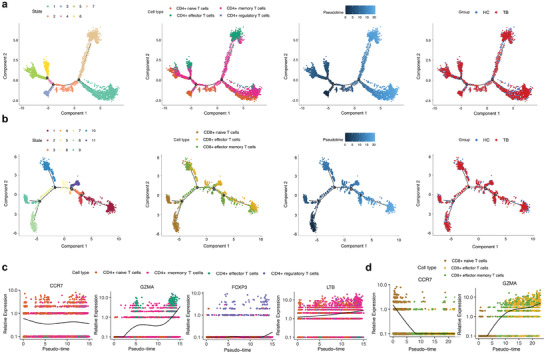
Developmental trajectories of CD4+ and CD8+ T cell subtypes and the expression pattern of representative genes. (a, b). Pseudotime analysis of CD4+ and CD8+ T cell subtypes colored by state, cell subtypes, pseudotime, and group, respectively. c, d). Expression pattern of representative genes (*CCR7*, *GZMA*, *FOXP3*, and *LTB*) in CD4+ T cells (c) and (*CCR7* and *GZMA*) in CD8+ T cells (d) along pseudotime development. Abbreviations: HC, healthy control; TB, tuberculosis.

Naive CD4+ T cells developed into memory cells, regulatory cells, or effector cells. Memory CD4+ T cells can also differentiate into effector T cells (**Figure** **4**a), with a trajectory that could be due to restimulation of MTB in our eight participants with a history of Bacillus Calmette–Guerin (BCG) vaccination. Naive CD8+T cells were located at branch 2. Effector T cells were mainly distributed at the ends of branches 1, 5, and 3, while effector memory T cells fell in between, indicating that this cell subtype was an intermediate form (Figure 4b).

In both subtypes, *CCR7* expression declined with cell differentiation, while the expression of other representative genes (*GZMA*, *FOXP3*, and *LTB*) increased (Figure 4c,d). These results suggest naive cells gradually acquired cytotoxicity, immunoregulatory, and memory functions and differentiated into other forms, through transcriptome reprogramming to some degree.^[^ ^15^ ^]^ However, beyond existing knowledge, the fact that there were no significant changes in the expression patterns of representative genes in these trajectories between TB and HC groups (Figure S4, Supporting Information) indicates that the process of functional transformation of T cells is not influenced in TB patients.

### 2.5 Decoding TB‐Associated Behaviors of Myeloid Cells

**Figure 5:**
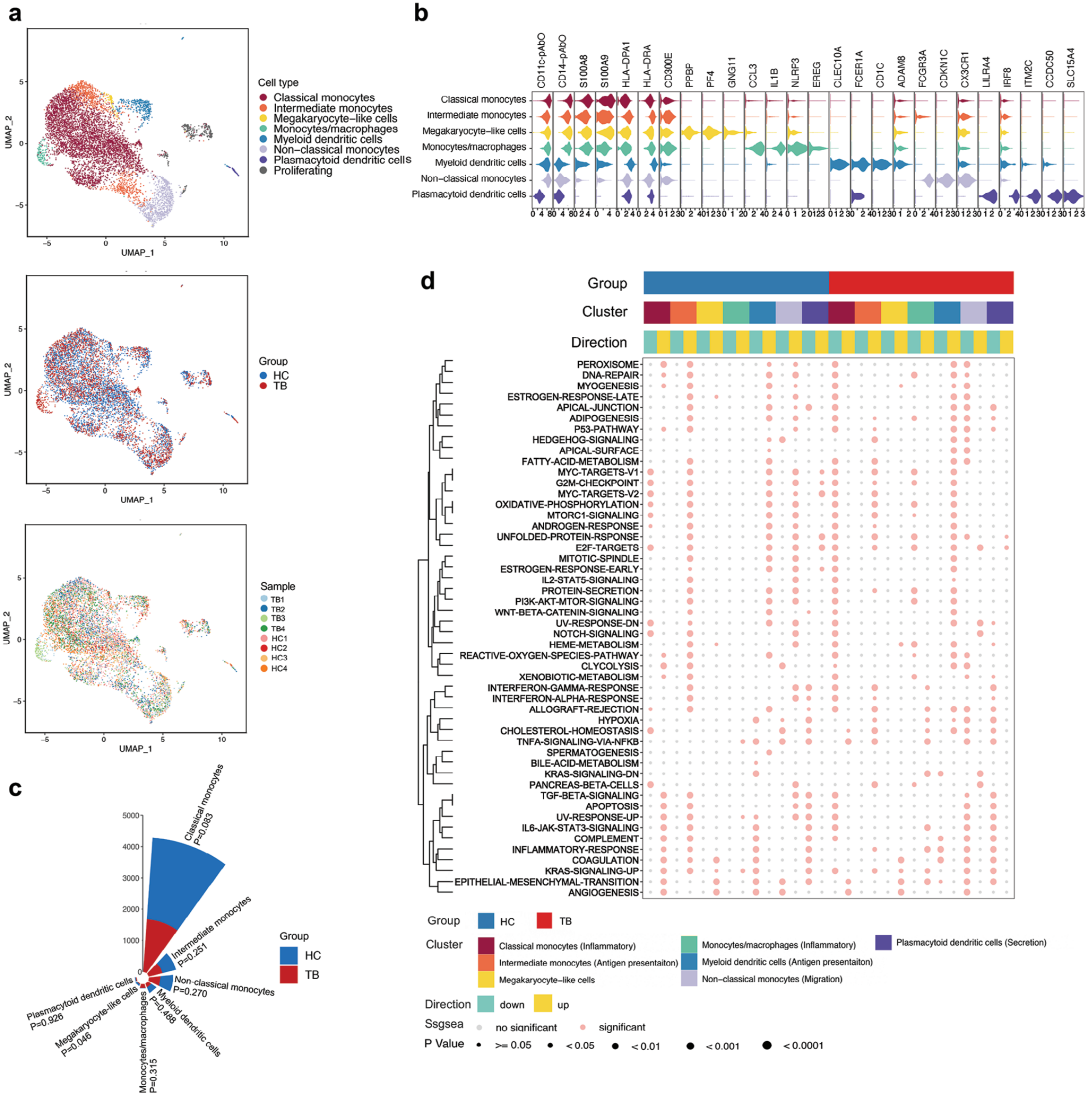
Cluster and cell subtype analysis of myeloid cells. a) UMAP plots of 6855 myeloid cells colored by cell type, group, and sample. b) Violin plots showing expression level for marker genes of seven myeloid cell subtypes. Molecules with names ending with pAbO represent cell surface proteins. c) Nightingale rose plot showing a number of seven myeloid cell subtypes colored by the group. *p* values are computed from a two‐sided Student's *t‐*test. d) Differences in ssGSEA score of hallmark top 50 gene sets in myeloid cell subtypes between the TB and HC groups. The color and size of the dots together indicate enrichment significance. *p* values are computed from the Wilcox test.

Myeloid cells have been reported as one of the predominantly altered cell types in TB, underlining their importance in pathogenesis.^[^ ^16^ ^]^ We performed a reclustering analysis of 6855 myeloid cells and grouped them into 16 clusters and eight subsets, including four subsets of monocytes, two subsets of dendritic cells, one subset of megakaryocyte‐like cells, and one subset of proliferating cells (**Figure** **5**a). Specifically, there were classical monocytes (*CD11c*, *CD14*, *S100A8*, and *S100A9*), intermediate monocytes (*HLA‐DPA1* and *HLA‐DRA*), nonclassical monocytes (*CDKN1C* and *FCGR3A*), monocytes/macrophages (*CCL3* and *IL1B*), myeloid dendritic cells (*CD16* and *CLEC10A*), plasmacytoid dendritic cells (*LILRA4*, *IRF8*, and *ITM2C*) and megakaryocyte‐like cells (*PPBP* and *PF4*) (Figure 5b).^[^ ^8^, ^17^ ^]^ The proportion of these myeloid cell subpopulations did not differ significantly between these two groups (Figure 5c). Functional variations were observed in different groups. For example, apoptosis, oxidative phosphorylation, and PI3K‐AKT‐mTOR signaling in classical monocytes of TB patients were suppressed (Figure 5d). The expressions of *STK17B*, *FCER1*, and other genes closely related to these pathways were significantly decreased in the TB group (Table S6, Supporting Information). These findings suggest the involvement of myeloid cells in TB, a finding which was also corroborated by La Manna MP et al.^[^ ^18^ ^]^

### 2.6 Characterization of B Cells and NK Cells

**Figure 6:**
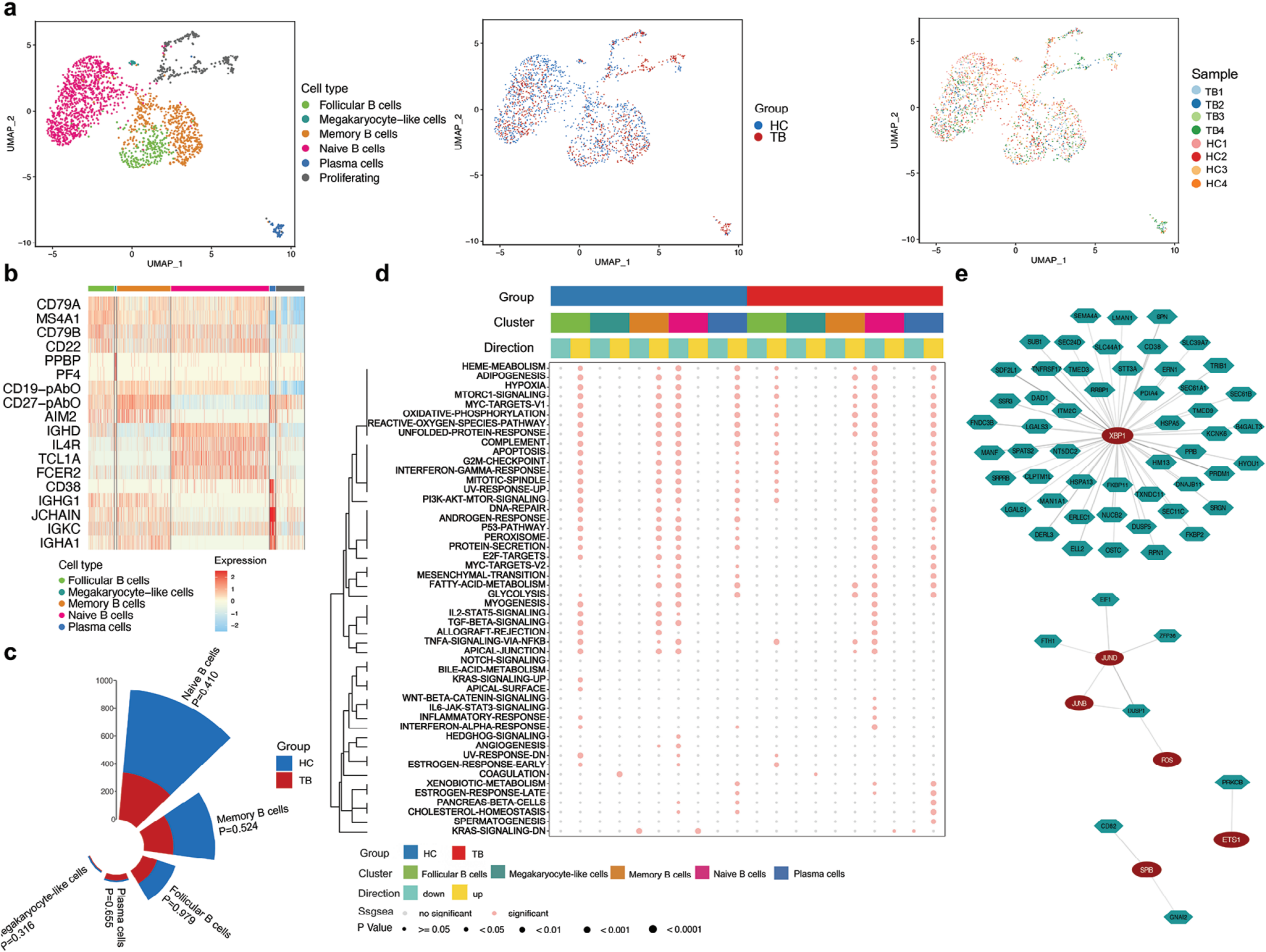
Cluster and cell subtype analysis of B cells. a) UMAP plots of 2035 B cells colored by cell type, group, and sample. b) Heatmap showing expression of marker genes of five B cell subtypes. Molecules with names ending with pAbO represented cell surface proteins. The red color denotes high expression, while the blue color denotes low expression. c) Nightingale rose plot showing a number of five B cell subtypes colored by the group. *p* values are computed from a two‐sided Student's *t*‐test. d) Differences in ssGSEA score of hallmark top 50 gene sets in B cell subtypes between TB and HC group. The color and size of the dots together indicate enrichment significance. *p* values are computed from the Wilcox test. e) Regulatory network of B cells, showing the pairs of regulatory factors (red circles) and genes (gene polygons) with a correlation>0.2 and high confidence. Abbreviations: HC, healthy control; TB, tuberculosis; ssGSEA, single‐cell rank‐based gene set enrichment analysis.

As previously shown, we found that the abundance of B cells was reduced in TB patients, consistent with the results of Joosten et al.^[^ ^19^ ^]^ We further reclustered 2035 B cells and grouped them into 6 subpopulations (**Figure** **6**a,b).^[^ ^8^, ^11^ ^]^ Naive B cells, memory B cells, and megakaryocyte‐like cells were the predominant cell subtypes that were decreased, although such differences were not statistically significant (Figure 6c). The signaling pathways always changed in the same direction for each B cell subtype in the same signaling pathways between TB and HC groups (Figure 6d). Many genes associated with these signaling pathways (*S100A9*, *CXCR4*, etc.) were differentially expressed between these two groups (Table S7, Supporting Information). These results hint at the fact that functional alternation of B cell subtypes could be related to their abundance rather than their transcriptomic characteristics, which was also observed in the nasopharyngeal carcinoma environment.^[^ ^20^ ^]^ As shown in the gene regulatory network, *XBP1* exhibited a more generic regulatory relationship than other genes and appeared to be a “hub gene.” *XBP1* is involved in the expression of 123 genes (Figure 6e). This TF was reported to be involved in B cell differentiation^[^ ^21^ ^]^ and is a promising target for controlling B cell differentiation.

**Figure 7:**
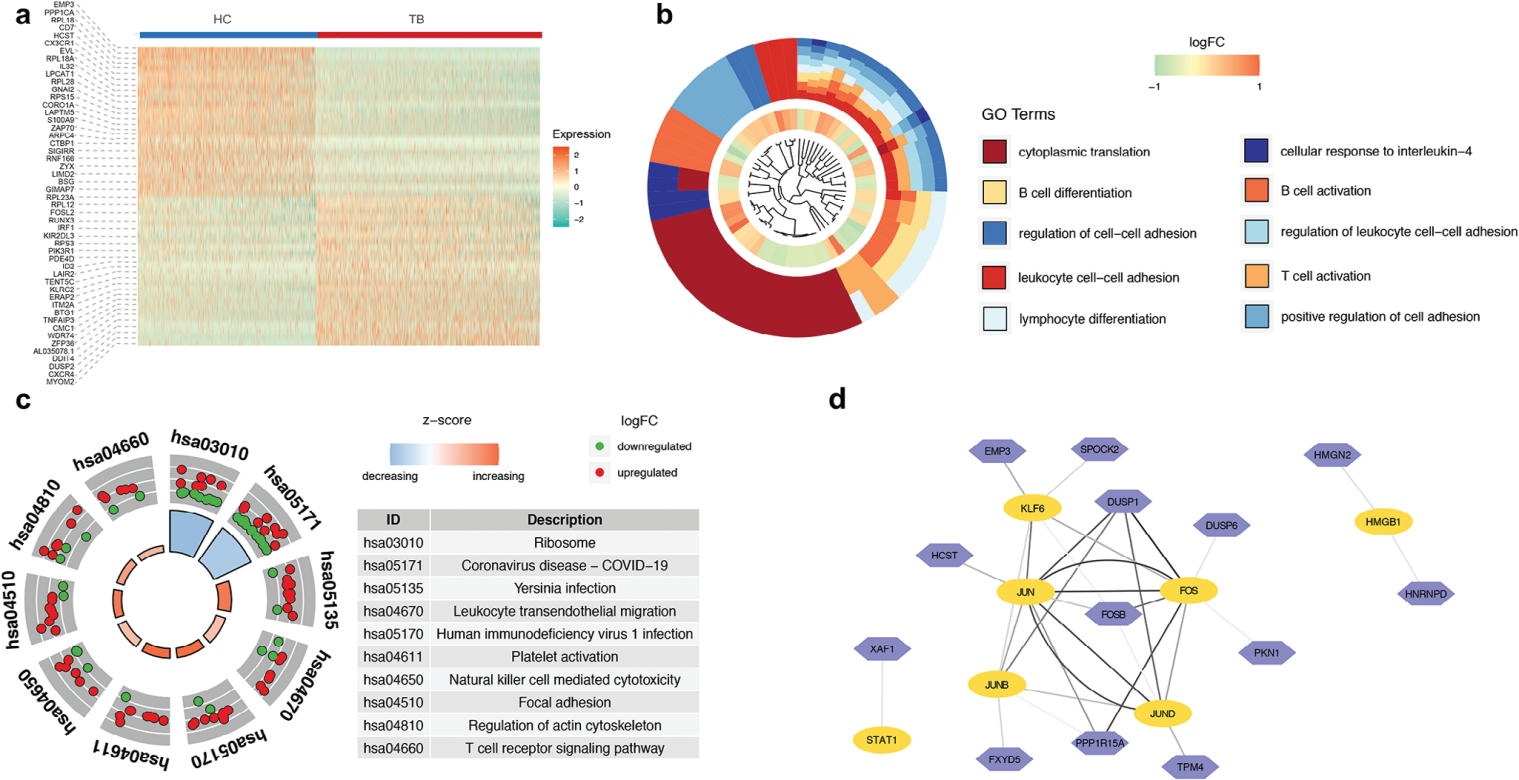
The transcription atlas of NK cells. a) Heatmap showing the most upregulated 25 genes and downregulated 25 genes of NK cells in TB and HC groups. b) GO cluster plot (top ten terms). The inner dendrogram represents the clustering according to the gene expression profiling. The middle circle represents the value of logFC, while the outer circle represents the enriched GO BP terms. c) GOCircle plot showing the result of KEGG analysis. Dots in the outer circle indicate genes related to the corresponding pathways. The colors of the inner circle indicate the z‐score. d) Regulatory network of NK cells, showing the pairs of regulatory factors (yellow circles) and genes (purple polygons) with a correlation>0.1 and high confidence. Abbreviations: NK, natural killer; HC, healthy control; TB, tuberculosis; GO, Gene Ontology; logFC, log2 fold change; BP, biological processes.

In total, 2451 and 1945 NK cells from TB patients and HCs were included. Transcriptional data showed that the TB group exhibited decreased *IRF1* and *MAP3K8* expression compared with the HC group (**Figure** **7**a). GO analysis indicated that these genes were mainly related to T cell and B cell activation, suggesting that MTB could indirectly affect other immune cells by targeting NK cells (Figure 7b), consistent with the result from a previous study.^[^ ^22^ ^]^ Besides, we find that some DEGs (e.g., *KLRC1* and *KLRC2*) were enriched into the pathways of NK cell cytotoxicity and leukocyte migration (Figure 7c), revealing the diverse and important roles of NK cells in TB. To gain more insight, we built a gene regulatory network and found that *DUSP1* was a co‐regulated gene of *JUN*, *JUNB*, *JUND*, and *FOS* (Figure 7d). *DUSP1* has been known to participate in mitogen‐activated protein kinase signaling pathway and inflammatory cytokines (TNF‐α, IL‐6, CCL3, and CCL4) release.^[^ ^23^ ^]^ Therefore, targeting the subunits of activator protein 1 to control *DUSP1* expression in NK cells might be a potential approach to regulate inflammation‐related reactions.

### 2.7 Identification of TB‐Related Cellular Communication

**Figure 8:**
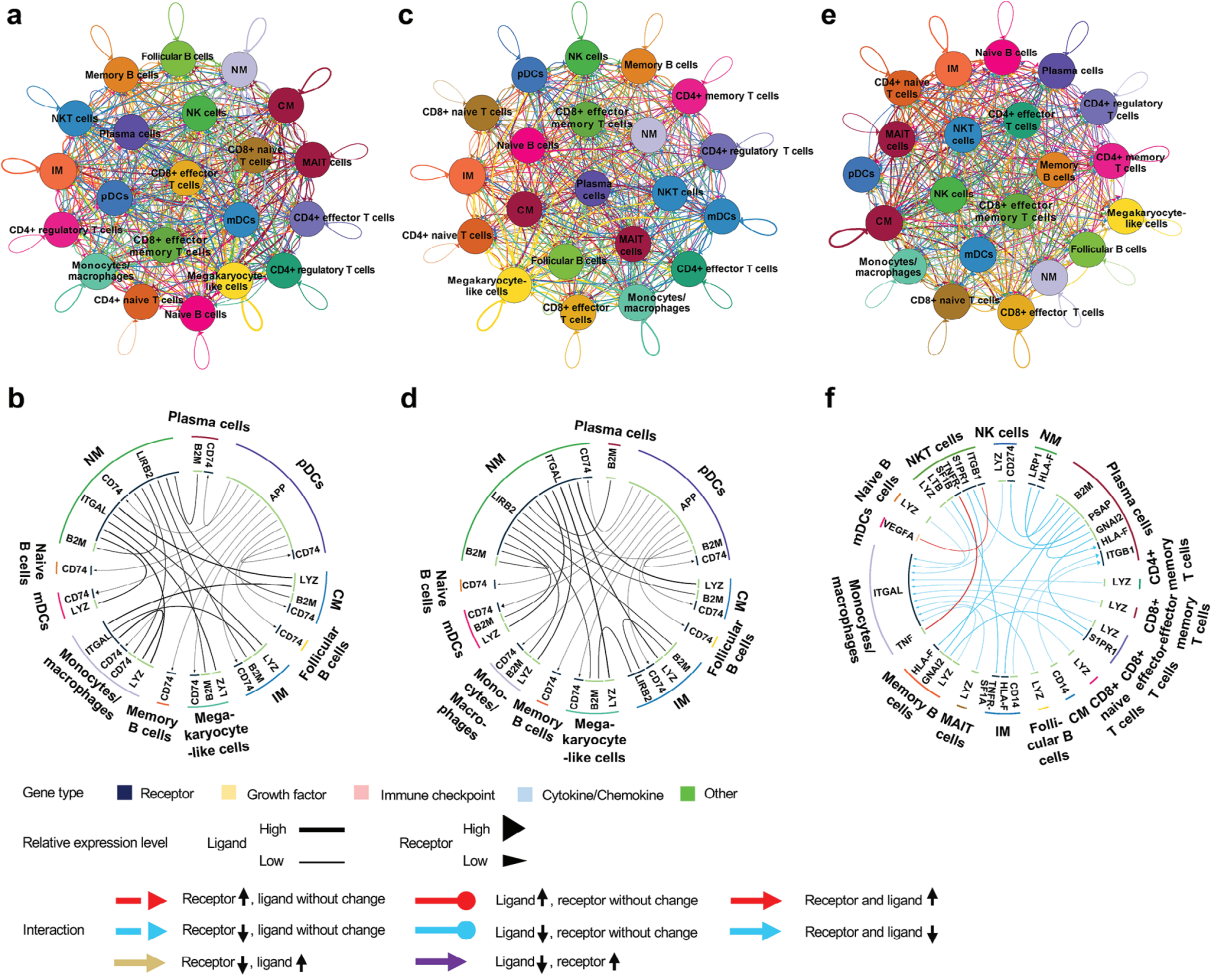
The network and circos plots of cellular communication. The network plots show the number of ligand–receptor pairs between two cell subtypes. In circos plots (top 25 pairs‐b, d, f), the outside ring represents cell subtypes, while the inside ring represents ligands or receptors. a, b) HC group; c, d) TB group; e, f) Differences between TB and HC groups. Abbreviations: CM, classical monocytes; IM, intermediate monocytes; NM, nonclassical monocytes; NK, natural killer; NKT, natural killer T; MAIT, mucosal‐associated invariant T.

We used iTalk to characterize cellular communication in TB and HC. In the HC group, intermediate monocytes interacted most frequently with other subtypes, whilst myeloid dendritic cells received the most interaction signals (**Figure** **8**a,b). In the TB group, megakaryocyte‐like cells sent and received the most interaction signals, while CD8+ naive cells had the least (Figure 8c,d).

Differential analysis (Figure 8e,f) showed that among all altered pairs, *VEGFA* and *ITGA9* were only responsible for communication between myeloid dendritic cells and CD4+ regulatory T cells. Increased *VEGFA* expression in the TB group enhanced the signaling of myeloid dendritic cells to CD4+ regulatory T cells (Table S8, Supporting Information). Both *VEGFA* and *ITGA9* have been found to be closely related to cell proliferation in the tumor environment.^[^ ^24^ ^]^ It could be inferred that the potential relationship between the altered myeloid dendritic cell‐CD4+ regulatory T cell communication by the *VEGFA*‐*ITGA9* pair and the imbalance of immunosuppressive and non‐immunosuppressive cells. The *VEGFA*‐*ITGA9* pair might serve as the potential target for correcting this immune imbalance in TB. Additionally, CD4+ regulatory T cells interacted with other T cell subpopulations mainly through *GNAI2* (Table S8, Supporting Information). The members of sphingosine‐1‐phosphate receptor (S1PR) (S1PR1, S1PR3, S1PR4, and S1PR5) were the major receptors that GNAI2 targeted. Both GNAI2 and S1PR members play critical roles in immune cell trafficking and cytokine production,^[^ ^25^ ^]^ such that decreased GNAI2 expression in the TB group could disturb immune cell trafficking and cytokine production.

## 3 Discussion

This work systemically studied TB‐related functional alterations of different cell subtypes in TB and characterized TB‐related immune function changes. Although there is existing evidence that despite a host being able to kill the MTB, it is known that during persistent re‐exposure in highly endemic settings, TB progression may still occur.^[^ ^26^ ^]^ We propose that understanding the mechanism of this failure in MTB intervention may be key to curbing the feedback between high public prevalence and individual TB progression. Amongst the cells MTB targeted in our detailed study, it is known that T cells contribute significantly to pathogen clearance and controlling the progression of infection.^[^ ^27^ ^]^ After stimulation, T cells, responsible for clearing pathogens, proliferate and differentiate into effector T cells and memory T cells (which ensures a rapid response upon restimulation). This pressure has led MTB to evolve an immune escape strategy against T cells, by inhibiting their function.^[^ ^28^ ^]^ Thus, although the host increases the abundance of cytotoxic cells to mount an immune defense, MTB gives continuous antigenic stimulation to induce cell dysfunction or exhaustion. This phenomenon was previously also observed in other infectious diseases and in patients who have not been treated.^[^ ^29^ ^]^ Our functional analysis further shows that the impaired T cell functions in the TB group are multidimensional and persistent.

Interestingly, the immunoregulation function of CD4+ regulatory T cells did not exhibit significant alternations between TB patients and HCs. CD4+ regulatory T cells with high levels of *FOXP3* expression have been reported to play an inhibitory role in effector responses via both a contact‐dependent and IL‐10 contact‐independent fashion.^[^ ^26^ ^]^ Cell–cell communication analysis implied that in TB, such a role might be realized by GNAI2 and S1PR members. GNAI2 is ubiquitously expressed in immune cells and participates in chemokine secretion and T cell and B cell trafficking.^[^ ^25^, ^30^ ^]^ S1PR members have also been shown to be closely associated with the trafficking and cytokine secretion in immune cells.^[^ ^31^ ^]^ Other receptors of GNAI2 identified in our study (CXCR1, CXCR2, CXCR3, and C5AR1) are also related to these processes.^[^ ^32^ ^]^ Although the interactions between these molecules and their effects have not been elucidated yet, the pathways involved imply that in TB patients, CD4+ regulatory T cells may inhibit T cell trafficking and cytokine secretion through GNAI2, thus further weakening the host immune strength.

This immunosuppressive state of the host leads to MTB proliferation, further exposing the host to enhanced antigenic stimulation and ultimately T cell exhaustion,^[^ ^33^ ^]^ as observed in our study through the upregulated expression of exhaustion‐related molecules (*Tim3*, *LAG3*, and *BTLA*) in T cell subtypes. This observation has the potential to suggest new therapeutic strategies, different from the current strategy of targeting the synthesis of MTB components, to interfere with replication or growth.^[^ ^34^ ^]^ We propose that in the future, by targeting exhaustion‐related molecules through new TB‐specific immunotherapy, it may be possible to rescue T cell dysfunction.

Compared with previous clinical TB‐related scRNA‐seq research, this work included a relatively large number of clinical cases, although this sample size was still limited. This is an initial clinical validation that goes beyond current work in preclinical models. Luckily, scRNA‐seq analysis is carried out based on cells, and therefore all analyses in this work are performed on the basis of 23 990 cells. Validations in both an independent scRNA‐seq dataset and bulk sequencing data also ensured the robustness of these findings. Furthermore, whether similar immune function changes exist in patients with latent MTB infection could not be assessed, due to the limited visits in this population and our strict inclusion criteria.

## 4 Conclusion

This work systemically studies TB‐related functional alterations of different cell subtypes and demonstrates the decreased naive, cytotoxicity, and memory functions of T cells in TB, that contributed positively to the host immune strength, rather than their immunoregulatory function. These findings, when taken together, allow us to detail the immune function changes of TB, which have the potential to generate new targets for novel immunotherapeutic treatment strategies to restore T cells from dysfunctional or exhausted states.

## 5 Experimental Section

### Participant Recruitment and Sample Collection

Two cohorts of individuals were enrolled. scRNA‐seq and AbSeq on samples from the first cohort was performed, to reveal TB‐related functional heterogeneity at the single‐cell level. Samples from the second cohort were subjected to bulk RNA sequencing to validate the findings from the first cohort. Individuals with primary TB were recruited from West China Hospital of Sichuan University between August 2021 and March 2022, with the following inclusion criteria, namely: i) as being diagnosed with TB based on Diagnostic Criteria for Tuberculosis (WS 288–2008)^[^ ^35^ ^]^; ii) as having received no anti‐TB treatment; iii) as having no previous TB or contact history; iv) as being aged 18 years or above; and v) with no other lung, liver, or autoimmune comorbidities. Pregnant women were excluded. Healthy controls (HCs) needed to meet the requirements of having no TB‐related features in chest computerized tomography scan, and no known history of TB history or contact, confirmed through negative results on IFN‐release assay.

PBMCs were isolated by density gradient centrifugation using Ficoll–Paque Plus medium (Cytiva) and cryopreserved in RPMI 1640 Medium (Gibco) containing 50% fetal bovine serum (Gibco) and 10% dimethyl sulfoxide (Beyotime) at −80 °C.

### Patient Consent and Ethics

This study was approved by the Clinical Trials and Biomedical Ethics Committee of West China Hospital, Sichuan University (2019‐829) (Registration number of Chinese Clinical Trial Registry: ChiCTR1900028670). Written informed consent was obtained from each included participant.

### ScRNA‐seq, AbSeq, and Bulk RNA Sequencing

The BD Rhapsody single‐cell analysis system was used to measure mRNA expression and surface proteins of single cells.^[^ ^36^ ^]^ Briefly, cryopreserved PBMCs were thawed and washed with Stain Buffer (BD Pharmingen). Calcein AM (BD Pharmingen) and DRAQ7 (BD Pharmingen) were applied to assess cell viability. Samples with cell viability greater than 90% and concentrations ≈1000 µL^−1^ were used. To differentiate the cell origin and detect cell surface proteins, cells were sequentially labeled by the single‐cell multiplexing kit (BD) and AbSeq immune discovery panel (BD), respectively. Labeled cells and beads were loaded into a cartridge read by the scanner (BD Rhapsody). After cell lysis, whole transcriptome mRNA, sample, and AbSeq tags were obtained and further bound to beads (Cartridge Reagent Kit, BD Rhapsody). Following reverse transcription (cDNA kit, BD Rhapsody), the extended AbSeq and sample tags were denatured from the beads and amplified by polymerase chain reaction (PCR) to obtain AbSeq and sample tags sequencing libraries. At the same time, the whole transcriptome mRNA library was generated from the beads by random priming and index PCR. After library amplification (WTA amplification kit, BD Rhapsody), sequencing was conducted on the NovaSeq 6000.

For bulk RNA‐seq, total RNA was extracted from PBMCs using TRIzol (ThermoFisher Scientific), and RNA quantity (>2 µg) and quality (RNA integrity number>6.5) were determined by Agilent 2100 Bioanalyzer. RNA fragmentation, purification and adaptor ligation, reverse transcription, and PCR amplification provided the library, sequenced by NovaSeq 6000.

Single‐cell sequencing data analysis is detailed in the Supporting Information File.

### Statistical Analysis

GraphPad Prism (v 7.0a) and R (v 4.1.2) were used. Functional marker gene expression was shown by heatmaps, while the function ability was expressed as mean ± standard deviation. Student's *t*‐test or Mann–Whitney test was used to calculate the *p‐*value, and the significance was set at *p* < 0.05 (two‐sided).

## Conflict of Interest

The authors declare no conflict of interest.

## Author Contributions

M.L., J.M.C., and B.Y. contributed to the conceptualization. M.L., G.X., and J.Z. were responsible for data curation. M.L., G.X., J.Z., J.R., Y.W., and H.L. investigated the data, chose the methodology, and conducted the formal analysis. Y.C. and Y.Z. visualized the data. J.M.C. and B.Y. administrated and supervised the whole project. B.Y. and J.M.C. provided the funding support. All the authors drafted, reviewed, and edited the manuscript.

## Supporting information

Supporting Information

Supporting Information

## Data Availability

The data that support the findings of this study are openly available in [China National Center for Bioinformation/Beijing Institute of Genomics, Chinese Academy of Sciences] at [https://ngdc.cncb.ac.cn/omix], reference number [1153].
