# Supplementary material for: Single‐Cell Sequencing Reveals Functional Alterations in Tuberculosis
Source: Adv Sci (Weinh). 2024 Jan 8;11(11):2305592. doi: 10.1002/advs.202305592 (PMC10953544; doi:10.1002/advs.202305592)
Supplement: Supplementary file 1 — Supporting Information [file ADVS-11-2305592-s002.pdf]

ADVANCED SCIENCE
Open Access

Supporting Information

for *Adv. Sci.*, DOI 10.1002/advs.202305592

Single-Cell Sequencing Reveals Functional Alterations in Tuberculosis

Mengyuan Lyu, Gaolian Xu, Jian Zhou, Julien Reboud, Yili Wang, Hongli Lai, Yi Chen, Yanbing Zhou, Guiying Zhu, Jonathan M. Cooper and Binwu Ying**

Single-cell sequencing reveals functional alterations in tuberculosis

Mengyuan Lyu¶, Gaolian Xu¶, Jian Zhou¶, Julien Reboud, Yili Wang, Hongli Lai，Yi Chen, Yanbing Zhou, Guiying Zhu, Jonathan M. Cooper, Binwu Ying**

* Corresponding author
Jonathan M. Cooper: Jon.Cooper@glasgow.ac.uk
Binwu Ying: yingbinwu@scu.edu.cn

This Supporting Information file includes:

(1) Supporting Methods-Data analysis

(2) Supporting Figures

Figure S1. Stacked bar chart showing proportion of included individuals in the discovery cohort.

Figure S2. Stacked bar charts showing cell proportions of the bulk sequencing cohort.

Figure S3. Expression of genes in functional gene sets (in rows) based on (in columns) the discovery cohort (a, d, g, j), an independent scRNA-seq dataset (b, e, h, k) and bulk data (c, f, i, l)

Figure S4. Expression pattern of representative genes in CD4+ T cell subtypes (a-*CCR7*, b-*GZMA*, c-*FOXP3* and d-*LTB*) and CD8+ T cell subtypes (e-*CCR7* and f-*GZMA*) along pseudotime development between TB and HC groups.

(3) Supporting Tables are supplied as .xlsx files.

Supporting Methods-Data analysis

ScRNA-seq and Ab-seq data analysis

Raw data processing

Raw readouts were processed according to the BD Rhapsody bioinformatics pipeline (https://bitbucket.org/CRSwDev/cwl/src/master/). After trimming, demultiplexing, and mapping to reference genome, gene and barcode matrices were obtained and further converted to a Seurat object by the Seurat package (v 4.0.5).

Cell clustering and annotation

Cells with feature counts >4000 or <500, or mitochondrial counts >15% were excluded. Gene matrices of each remaining cell were normalized with dimensional reduction based on 2000 variable genes. Subsequently, cells from different samples were combined with integration anchors, and after data scaling, both principal component analysis (PCA) and uniform manifold approximation and projection (UMAP) were used to reduce dimensionality, creating cell clustering.

Clusters were annotated as different cell types by both the SingleR package (v 1.8.0) and manual retrieval, based on well-labelled marker genes. Cells that expressed marker genes of different cell types were defined as proliferating cells and further excluded from the following analysis. Dimensional reduction plots, violin plots or heatmaps were drawn to visualize marker genes for each cell type.

Differential gene expression and signaling pathways analysis

For each cell type, differentially expressed genes (DEGs) between TB patients and HCs were identified based on the Wilcoxon Rank Sum test. Genes with Bonferroni-adjusted p value <0.05 were considered to be significantly differentially expressed. The heatmap was plotted to present the expression of these DEGs in specific cell clusters between TB patients and HCs.

The integration of single-cell rank-based gene set enrichment analysis (ssGSEA) was carried out on the irGSEA package (v1.1.2), to assess the differentially up- or down-regulated gene sets of specific cell clusters between TB patients and HCs. For significant DEGs in some specific cell types, Gene Ontology (GO) and Kyoto Encyclopaedia of Genes and Genomes (KEGG) analyses were also performed by clusterProfiler package (v 4.2.1) to identify enriched pathways. Bubble plots, heatmaps and circle plots were used to exhibit the enrichment results.

Quantitative assessment of T cell function

According to T cell subtypes, 4 functions (naive, cytotoxicity, immunoregulation and memory) were presented. Representative cell subtypes of each function and their representative gene were selected. For example, CD4+ regulatory T cells were the representative cells of immunoregulation function, and *FOXP3* was the representative gene of this function. The system of quantitative functional assessment was constructed as previously described.[1] Briefly, the Spearman correlation coefficients of other genes and each representative gene were calculated, and the most related 10 genes were further selected to construct functional gene sets. A linear model was applied to integrate the selected genes, and the probability generated by the linear model was used to assess functional ability (stats package, v 4.1.2).

Pseudotime analysis

Monocle2 package (v 2.22.0) was used to order cells in pseudotime and infer the trajectory of cell differentiation. After quality control, the filtered genes were input into the reversed graph embedding, and cell differentiation process was inferred from gene expression patterns. Trajectory trees were plotted to visualize the above process.

Gene regulatory network construction

Gene regulatory network analysis (using the SCENIC package, v 1.2.4) was based on the correlation between transcription factors (TFs) and each gene, followed by the formatting and filtering co-expression modules, motif enrichment analysis and generating regulons. TFs with top 5 weights for each gene and high confidence entered further analysis. The threshold of correlation was flexibly defined according to the number of pairs, and the gene regulatory network was shown by Cytoscape (v 3.7.2).

Cellular communication

Intercellular communication was investigated with the iTALK package (v 0.1.0). Although only cells in the same sample can communicate with each other, we took groups (TB group or HC group) as units for this analysis to overcome the error caused by the sparsity of single-cell data. Cellular communication signals were reported according to the expression pattern of ligands and receptors and their potential relationships. Differences in cellular communication between TB and HC groups were determined based on DEGs identified by DESeq2. The circle plots were generated to show ligand-receptor pairs.

Bulk sequencing data analysis

Raw reads with length <36bp or quality score <25 were discarded and adaptors were trimmed by trim galore (v 0.6.7). Hisat2 (v 2.1.0) was used to align clean reads to the reference genome (GRCh38), while featureCounts (v 2.0.3) was applied to count reads.

TB-related sequencing datasets acquisition and processing

To improve the reliability of the results, we re-analyzed public TB-related scRNA-seq data to validate TB-specific functional changes in various cell subpopulations, in addition to our validation at bulk level. TB-related sequencing datasets were retrieved in January 2022 from NCBI Gene Expression Omnibus database (GEO), NCBI Sequence Read Archive (SRA) and European Bioinformatics Institute (EBI) ArrayExpress. The search terms were ("tuberculosis" OR "TB"), while the filters involved the detection methods (sequencing), species (homo species) and sample type (PBMC). Data generated from in vitro models were excluded.

A scRNA-seq dataset (SRA ID: SRP247583) generated from 10 genomes was included. Raw SRA files of TB patients and HCs in this dataset were downloaded and converted into fastq files. CellRanger (v 6.1.2) was employed to generate gene and barcode matrices and subsequent analysis was described in the Cell clustering and Annotation section.

For bulk RNA sequencing, the raw file of each sample was downloaded and processed according to the Bulk sequencing data analysis section. After being counted, all data from different datasets were

merged, and the average value was computed as the count of the genes detected repeatedly. Subsequently, sva package (v 3.42.0) was used to adjust the batch effect among different datasets.

Bulk sequencing data deconvolution

CIBERSORTx (https://cibersortx.stanford.edu/) was applied to deconvolute the bulk sequencing data. A total of 268 marker genes of 21 cell subpopulations in our scRNA-seq data were selected and 10% cells were randomly extracted to generate the signature matrix. Based on the above matrix, the bulk sequencing data was deconvoluted and the cell abundance for each sample was acquired. We also divided the samples into different groups to obtain cell-specific gene expression.

References

[1] L. Gong, D. L. Kwong, W. Dai, P. Wu, S. Li, Q. Yan, Y. Zhang, B. Zhang, X. Fang, L. Liu, M. Luo, B. Liu, L. K. Chow, Q. Chen, J. Huang, V. H. Lee, K. O. Lam, A. W. Lo, Z. Chen, Y. Wang, A. W. Lee, X. Y. Guan, *Nature communications* **2021**, 12, 1540.

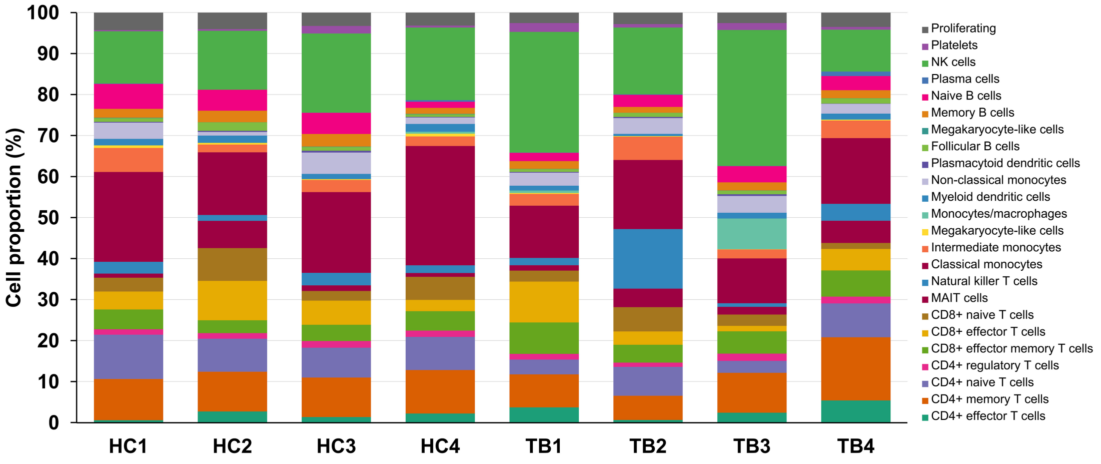

Figure S1. Stacked bar chart showing proportion of included individuals in the discovery cohort. **Abbreviations:** TB: tuberculosis; HC: healthy control; MAIT: mucosal associated invariant T cells; NK: natural killer

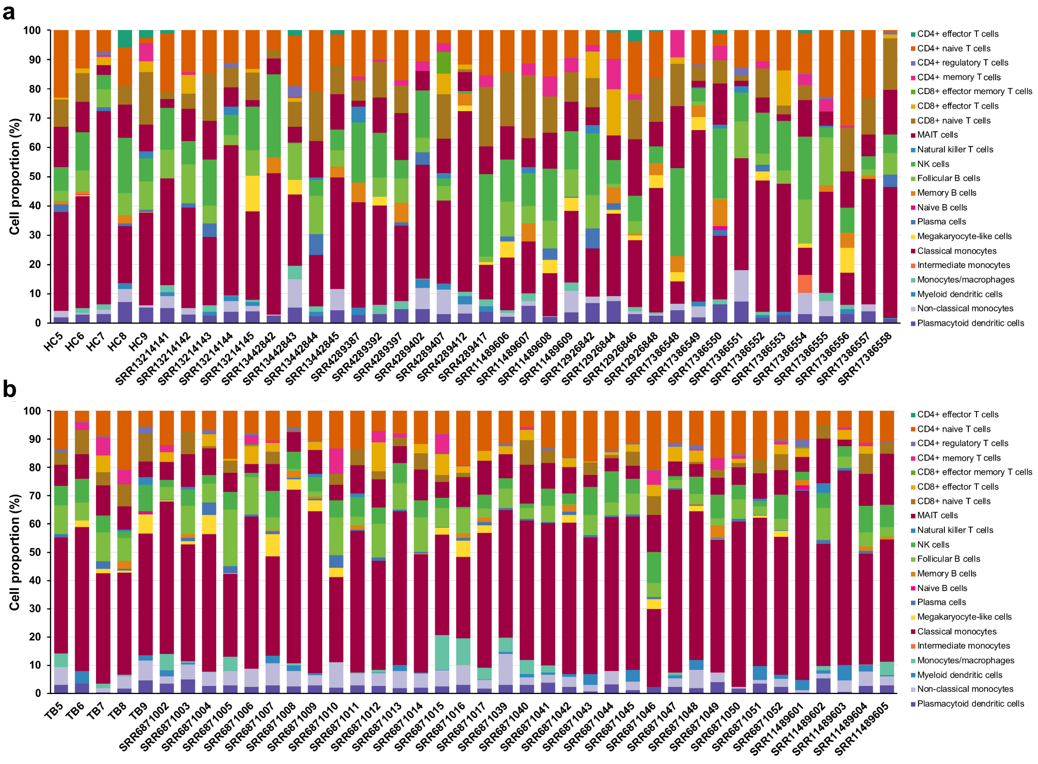

Figure S2. Stacked bar charts showing cell proportions of the bulk sequencing cohort. **a.** Cell proportion of sample in healthy control group. **b.** Cell proportion of sample in tuberculosis group. CD4+ regulatory T cells have been shown to exert a negative effect on host immunity to maintain immune balance and prevent immune injury. **Abbreviations:** MAIT: mucosal associated invariant T cells; NK: natural killer

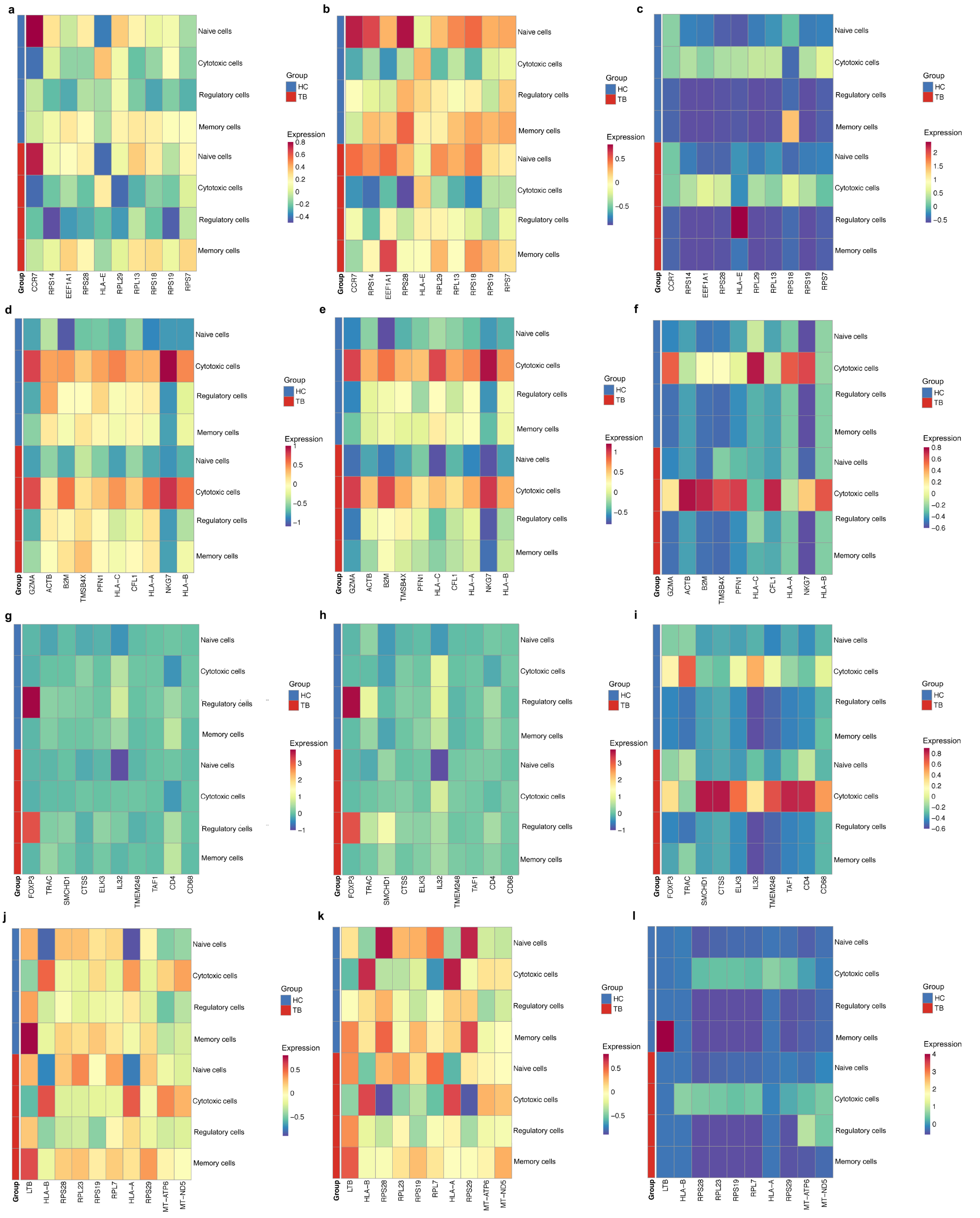

Figure S3. Expression of genes in functional gene sets (in rows) based on (in columns) the discovery cohort (a, d, g, j), an independent scRNA-seq dataset (b, e, h, k) and bulk data (c, f, i, l). **a-c** naive functional gene set. **d-f** cytotoxicity functional gene set. **g-i** immunoregulatory functional gene set. **j-l** Expression of genes in the memory functional gene set. **Abbreviations:** TB: tuberculosis; HC: healthy control

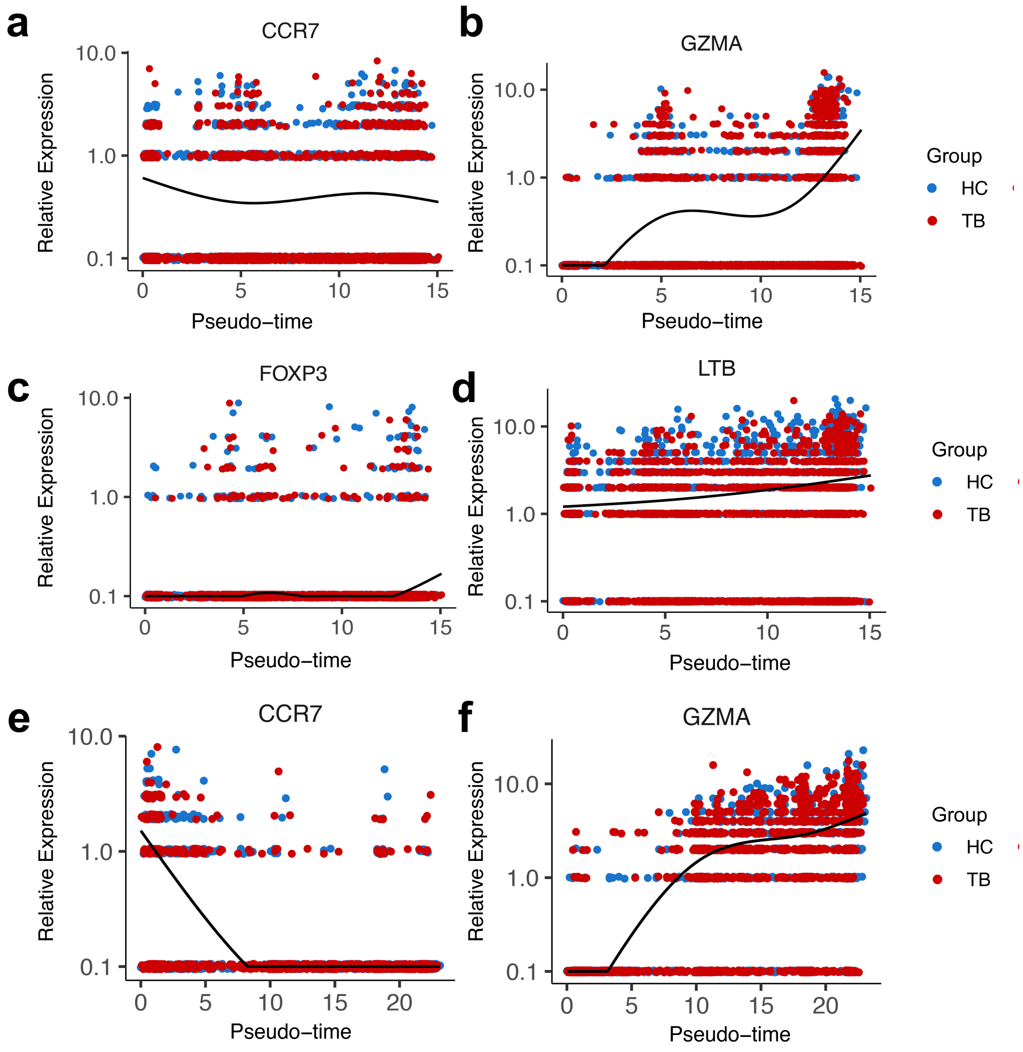

Figure S4. Expression pattern of representative genes in CD4+ T cell subtypes (a-*CCR7*, b-*GZMA*, c-*FOXP3* and d-*LTB*) and CD8+ T cell subtypes (e-*CCR7* and f-*GZMA*) along pseudotime development between TB and HC groups. **Abbreviations:** HC: healthy control; TB: tuberculosis.
